# Rewinding the Clock: Emerging Pharmacological Strategies for Human Anti-Aging Therapy

**DOI:** 10.3390/ijms26199372

**Published:** 2025-09-25

**Authors:** Charlotte Delrue, Reinhart Speeckaert, Marijn M. Speeckaert

**Affiliations:** 1 Department of Nephrology, Ghent University Hospital, 9000 Ghent, Belgium; charlotte.delrue@ugent.be; 2 Department of Dermatology, Ghent University Hospital, 9000 Ghent, Belgium; reinhart.speeckaert@uzgent.be; 3 Research Foundation-Flanders (FWO), 1000 Brussels, Belgium

**Keywords:** aging, senolytics, NAD^+^ precursors, metformin, mTOR inhibitors, AMPK activators

## Abstract

Aging is a complex, multifactorial process characterized by progressive physiological decline and increased vulnerability to chronic diseases and syndromes. Recent studies have highlighted nine interrelated hallmarks of aging, emerging primarily from an understanding of cellular homeostasis, health, and senescence, such as genomic instability, telomere attrition, and cellular senescence. These hallmarks provide a conceptual framework for advancing pharmacotherapeutic interventions. In this review, we present an overview of old and new pharmacotherapeutic interventions that are being developed using these hallmarks of aging to enhance healthspan delay and ameliorate age-related pathologies. We classify these strategies into five broad categories, including senolytics, senomorphics, NAD^+^ precursors, mTOR inhibitors, and metabolic modifiers, such as metformin, and review the mechanisms by which they act, preclinical evidence for efficacy, and their translational potential to a clinical context. In addition, we consider the clinical landscape and report the important trials that are currently underway and some of the main obstacles, including key challenges such as biomarker identification, safety issues, and regulatory challenges. Overall, we discuss ahead-of-time gerotherapeutics and the important role of a collective, mechanism-focused basis for therapeutically targeting aging biology.

## 1. Introduction

**Figure 1:**
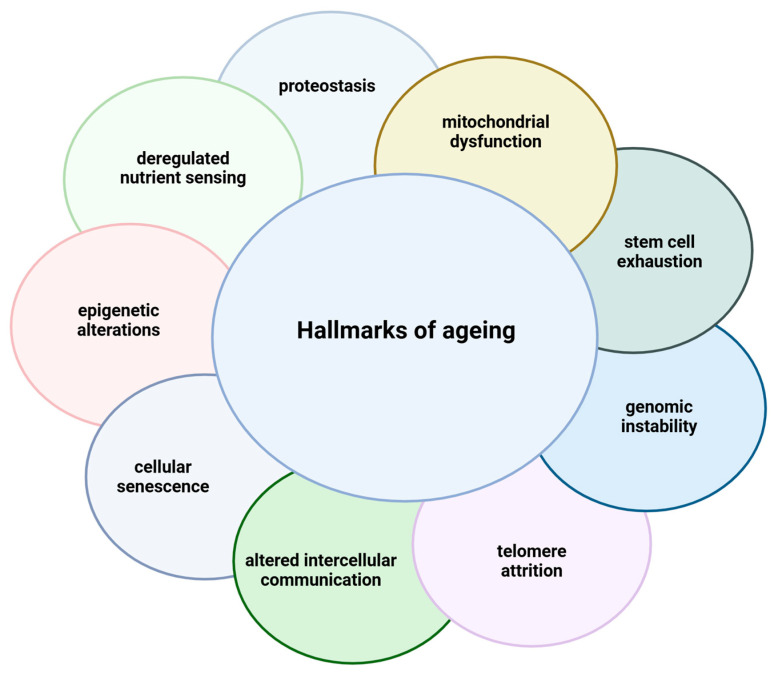
Illustration of the nine hallmarks of aging, each represented by a distinct colored circle surrounding the central concept. Proteostasis denotes the age-related decline in the cell’s capacity to maintain normal protein folding and degradation, resulting in the accumulation of damaged proteins. Mitochondrial dysfunction includes the age-related decline in mitochondrial function and an increase in deleterious reactive oxygen species. Deregulated nutrient sensing represents the failure of important pathways in energy metabolism, including insulin/IGF-1, AMPK, and mTOR, which can lead to a reversal of the energetic balance in the cell. Epigenetic alterations refer to changes in DNA methylation, histone modifications, and chromatin remodeling that affect gene expression in older organisms. Cellular senescence describes permanent cell division arrest accompanied by the release of inflammatory mediators, known as the senescence-associated secretory phenotype (SASP). Altered intercellular communication accounts for chronic low-grade inflammation (“inflammaging”) and impaired intercellular communication that arises with age. Telomere attrition is the accumulation of reductions at the ends of chromosomes, which limits the cellular replicative potential. Genomic instability is caused by the accumulation of DNA damage and mutations, which compromises genetic integrity. Lastly, stem cell exhaustion describes the lack of regenerative capacity of tissues due to defective stem cell replenishment.

Aging is a biological transition that results in a gradual decline in physiological and cellular functions. The aging process results in a decrease in function and an increase in vulnerability to chronic diseases, such as neurodegenerative diseases, cardiovascular diseases, type 2 diabetes, and cancer. The hallmark characteristics of aging cells are loss of proteostasis, mitochondrial dysfunction, deregulated nutrient sensing, genomic instability, telomere attrition, epigenetic alterations, cellular senescence, stem cell exhaustion, and altered intercellular communication. These nine hallmarks of aging are correlated and provide a solid basis for studying aging biology, offering numerous opportunities for pharmacological intervention (Figure 1) [1]. In this context, ascorbic acid (AA, also known as vitamin C) is a promising candidate due to its antioxidant and mitochondrial effects. While many studies have assessed AA in combination with other antioxidants or dietary interventions, the novelty of examining AA in isolation deserves emphasis, as it allows for a clearer attribution of potential neuroprotective and geroprotective properties to this single compound [2,3].

From a clinical practice perspective, the aging population is changing the delivery of healthcare services globally. The World Health Organization (WHO) predicted that by 2050, 2 billion men and women will be 60 years or older, which would double the number of older persons who were 60 years or older in 2020. This population shift has increased the demand for chronic disease management, long-term care, and rational drug use in older adults [4].

Effective interventions are needed to address the challenges confronting health systems arising from multimorbidity, polypharmacy, and functional decline in the older population. The negative impact of aging has large social and economic costs. Age-related diseases are among the leading causes of disability-adjusted life years (DALYs) and global health expenditure. In high-income countries, 50–60% of healthcare expenditure is for those aged ≥ 65 years [5]. In relation to the increase in life expectancy in populations, healthy life expectancy (HALE) has not advanced at the same pace. Consequently, there is a growing need to shift from a paradigm of disease-related care to one that supports healthspan; that is, the period of life lived in good health without chronic disease and disability. This model encourages us to take ownership of our health and seek platforms that can help us extend our healthspan while optimizing function, resilience, and independence, rather than lifespan [6,7,8].

Interest in pharmacological strategies targeting aging has increased significantly over the past 20 years. Geroscience has emerged as a new field in life extension science. It focuses on targeting the biological mechanisms of aging to avoid or delay age-related diseases. Key drug compounds, including metformin, rapamycin, NAD^+^ precursors, and senolytic compounds, have been shown to extend lifespan and/or healthspan in various model organisms [1]. Other candidates include senomorphics, AMPK activators, and CD38 inhibitors, which regulate inflammation, metabolism, and cellular senescence.

Despite increasing interest in the field and the number of preclinical and clinical studies, many studies vary in quality, model system, endpoints, and even definitions of “aging” and “healthspan.” Furthermore, one barrier is the exclusion of “older adults” in human studies because of comorbidities and polypharmacy, which limits the generalizability of the findings [9]. Other barriers include a lack of women, ethnic minorities, and socio-economically disadvantaged groups in many studies. Given the scale and urgency of the aging crisis, we provide a coherent, accessible narrative review of the current landscape of anti-aging drugs, what works, what does not, and what is next.

## 2. Hallmarks of Aging: Pharmacological Targets

Pharmacological drugs targeting the hallmarks of aging could offer an important means of extending the healthspan and postponing age-associated diseases. The first hallmark, genomic instability, is the accumulation of DNA damage caused by endogenous stress, e.g., reactive oxygen species (ROS) and exogenous agents (e.g., radiation and chemicals). Genomic instability alters the genome through pathways that promote malignant transformation and cellular senescence. Several strategies have been explored as potential countermeasures to mitigate genome damage. Poly(ADP-ribose) polymerase (PARP) inhibitors have been studied for their anticarcinogenic effects and potential to enhance DNA repair in normal tissues. However, the propensity of PARP inhibitors to have off-target effects, in addition to interfering with senescent responses that suppress tumor growth, raises safety concerns in healthy individuals. Metformin can also reduce oxidative DNA damage indirectly by improving mitochondrial function and reducing ROS production. Although observational studies have shown a decrease in cancer incidence in patients taking metformin, there are no robust randomized controlled trials (RCTs) involving healthy populations. Sirtuin (SIRT)-activating compounds (STACs), such as SRT1720, enhance DNA repair pathways via SIRT1 signaling, maintaining genomic stability [10]. However, there have been difficulties in converting STACs to clinical use because of their low bioavailability and uncertain safety.

Telomere attrition is the progressive shortening of telomeres associated with cell division, which is well defined in human biology and contributes to replicative senescence. A few pharmacological options are available to maintain or elongate telomeres. Telomerase activators are an example of such compounds. Agents such as TA-65, an extract from the Chinese herb Astragalus membranaceus (Mongolian milk vetch), may elongate telomeres in the immune cells. However, these effects have not yet been validated or tested clinically in the geriatric population. Danazol, a synthetic androgen, stimulates hematopoietic stem cell telomerase expression. However, this raises safety concerns because of potential androgenic side effects and unknown risks associated with TA-65. NAD^+^ precursors and caloric restriction mimetics may also help preserve telomeres by mitigating oxidative stress and enhancing cellular respiration to improve mitochondrial function [11]. However, human data in this field are still preliminary.

Epigenetic modifications, including DNA methylation, histone modifications, and chromatin structure, can accumulate over time and modify gene expression. Gene expression can be reprogrammed epigenetically, and many historical pharmacological agents that have been used to reprogram genes are histone deacetylase (HDAC) inhibitors (i.e., valproic acid, trichostatin A), which may be able to reinstate a younger epigenetic regulatory state. There are also DNA methyltransferase (DNMT) inhibitors (i.e., 5-azacytidine), which can reprogram gene expression. However, the approval of pharmacological agents has been restricted, mostly for oncology indications, while in non-diseased tissues, they have often proven to be toxic and limited. The transient expression of Yamanaka transcription factors (OSKM: Oct4, Sox2, Klf4 and c-Myc) has produced evidence of partial epigenetic reprogramming with the reversal of markers of aging in animal models. However, concerns regarding teratogenicity and oncogenic transformation limit their therapeutic applications in the near future [12].

Misfolded proteins linked to aging and aging-related illnesses, including Parkinson’s and Alzheimer’s diseases, typically accumulate and aggregate due to disturbed proteostasis or protein homeostasis. By blocking mechanistic target of rapamycin (mTOR) signaling, small-molecule medications may offer a way to increase autophagy and remove aging-related protein aggregates while reestablishing autophagy flux. For example, mTOR inhibitors (rapamycin and rapalogs) promote aggregate clearance. Other small molecules of interest are heat-shock protein (HSP) inducers, such as geranylgeranylacetone, and naturally derived polyamines (e.g., spermidine), which have been shown to restore autophagic flux and subsequently, proteostasis in aged tissues [13]. Although a fair amount of evidence has been provided by in vivo and in vitro studies, the human literature is still developing.

Deregulated nutrient sensing indicates improper signaling through the insulin/insulin growth factor (IGF)-1 pathway, AMP-activated protein kinase (AMPK), mTOR, and sirtuins. All these signaling pathways are linked to caloric intake and energy metabolism. For example, metformin (an AMPK activator) functions similarly to caloric restriction by increasing insulin sensitivity, whereas rapamycin inhibits mTOR with a mean lifespan extension in several mouse studies. Studies on NAD^+^ precursors, such as nicotinamide mononucleotide (NMN) and nicotinamide riboside (NR), have reported that they restore mitochondrial metabolism and trigger sirtuin signaling, which are beneficial for metabolic health and lifespan [14]. However, while these compounds are generally well tolerated in the short term, their long-term safety and efficacy in aging have yet to be established, and high doses of NAD^+^ compounds may disrupt methylation balance.

Mitochondrial dysfunction is another characteristic of maturation, resulting in reduced energy production and increased levels of oxidative stress. Many pharmaceutical approaches to restore mitochondrial function exist, the first being the use of mitochondria-targeted antioxidants, such as Mitoquinone (MitoQ) and 10-(6′-plastoquinonyl) decyltriphenylphosphonium (SkQ1), which preferentially accumulate in the mitochondria targeting ROS and apoptosis. Urolithin A, derived from ellagitannins in the gut microbiome, induces mitophagy and mitochondrial biogenesis in the host. Preliminary human trials using Urolithin A in older adults have indicated improvements in muscle endurance, suggesting possible translation. NAD^+^ boosters are also directly related to maintaining mitochondrial integrity by propelling oxidative and redox homeostasis, and mitochondrial DNA repair [15].

Cellular senescence drives chronic inflammation and tissue dysfunction via the senescence-associated secretory phenotype (SASP). Dasatinib (a tyrosine kinase inhibitor) and quercetin (a flavonoid) are senolytic drugs used to target and eliminate senescent cells. Fisetin, a natural and senolytic compound, has been found to act favorably in mouse models of aging. Senomorphics, including rapamycin and metformin, modulate SASP instead of removing senescent cells [10].

Stem cell exhaustion is defined as a progressive decline in the regenerative capacity of a tissue due to the loss and/or dysfunction of adult stem cells. Pharmacological methods to stimulate stem cell pools for renewal include hematopoietic-stimulating agents such as granulocyte colony-stimulating factor (G-CSF) and erythropoietin (EPO). Resveratrol (polyphenol) and NAD^+^ precursors or boosters can provide similar benefits for stem cell health by enhancing mitochondrial health and decreasing oxidative injury, respectively. However, there is little evidence in humans, and stem cell-based therapy remains experimental. However, safety and immunogenicity issues and regulatory barriers hamper this effort [16].

Finally, alterations in intercellular communication, particularly chronic low-grade inflammation (“inflammaging”), are considered hallmarks of systemic aging. Janus kinase (JAK) inhibitors, such as ruxolitinib, inhibit inflammatory signaling cascades in cells. Anti-inflammatory agents, such as low-dose aspirin and interleukin (IL)-6 blockers, have been considered to prevent or reduce the effects of “inflammaging”. Changing our gut microbiota through prebiotics, probiotics, and diet could potentially improve healthy intercellular signaling and improve the immune response [17]. However, long-term interruption of immune signaling can increase the risk of infections, and the response to improved microbiome or other dietary interventions is very heterogeneous among different individuals.

**Figure 2:**
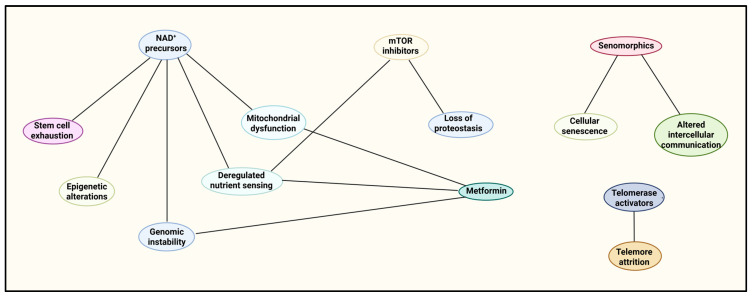
Pharmacological strategies mapped to the hallmarks of aging. This network diagram illustrates the relationships between several classes of pharmacological interventions and the specific hallmarks of aging they influence. Each oval represents either a drug class or a hallmark, with direct connections indicated by black lines. The hallmarks are represented across the network without explicit grouping by background, but they correspond to recognized domains: genomic stability (genomic instability, telomere attrition, epigenetic alterations), proteostasis and metabolic regulation (loss of proteostasis, deregulated nutrient sensing, mitochondrial dysfunction), and cellular/systemic integrity (cellular senescence, stem cell exhaustion, altered intercellular communication). NAD^+^ precursors are connected to multiple hallmarks, including mitochondrial dysfunction, genomic instability, stem cell exhaustion, deregulated nutrient sensing, and epigenetic alterations, reflecting their broad impact on cellular repair, energy metabolism, and epigenetic regulation. mTOR inhibitors are linked to loss of proteostasis and deregulated nutrient sensing, indicating their role in restoring protein homeostasis and nutrient-sensing pathways. Metformin is connected to deregulated nutrient sensing, mitochondrial dysfunction, and genomic instability, consistent with its known effects on metabolic signaling, mitochondrial efficiency, and oxidative stress. Senomorphics target cellular senescence and altered intercellular communication, modulating the senescence-associated secretory phenotype (SASP) and inflammatory signaling. Telomerase activators are linked exclusively to telomere attrition, reflecting their specific role in telomere length maintenance.

Drug interventions for each of the hallmarks of aging provide a potential route to delay functional decline and, therefore, increase the healthspan (Figure 2). Individual agents may offer unique advantages. However, the interconnected nature of these hallmarks suggests that a combination or sequence of therapies may be required for total rejuvenation.

## 3. Key Anti-Aging Drug Classes and Mechanisms

### 3.1. Senolytics: Dasatinib, Quercetin, and Fisetin

Cellular senescence may be induced by several factors, including telomere shortening, genotoxic stress, or anything that increases oncogene activity or mitochondrial malfunction. Senescence is a protective mechanism that results from tissue repair or, more accurately, the suppression of malignancy. Over decades, the aggregation of senescent cells disturbs tissue homeostasis quickly by the senescence-associated secretory phenotype (SASP) and its pro-inflammatory, pro-fibrotic, and proteolytic measures, such as IL-6, IL-1β, tumor necrosis factor (TNF)-α, matrix metalloproteinases (MMPs), and chemokines [18]. Senescent cells are at the heart of chronic “inflammaging,” observed in several aging-associated diseases and conditions, such as atherosclerosis, type 2 diabetes, sarcopenia, osteopenia, some neurodegenerative diseases, and cancer [19]. Senescent cells are most commonly identified using several features, including increased expression of p16^INK4a^ and p21^CIP1^, increased activity of senescence-associated β-galactosidase (SA-β-Gal), the presence of components that reveal DNA damage foci measured using γ-H2AX, and the accumulation of lipofuscin granules competing for oxygen in the tissue [20].

Senolytics are pharmacologic agents that selectively induce apoptosis of senescent cells by interfering with the overactive senescence-associated pro-survival signaling pathways [SASP(s)] that are upregulated in senescent cells. The SASPs modulated by them include B-cell lymphoma 2 (BCL-2) family proteins, phosphoinositide 3-kinase/protein kinase B (PI3K/AKT), the p53/p21^CIP1^ signaling pathway, and various tyrosine kinase-mediated pro-survival signaling cascades. By inhibiting the senescent cell pro-survival networks, senolytics can overcome the intrinsic apoptosis resistance in senescent cells while also acting on healthy proliferating and quiescent cells [18]. Importantly, senescent cells are preferentially removed from tissues via immune clearance, primarily dependent on natural killer (NK) cells and macrophages, which are lost with age [21]. The principal agents under investigation for senolytic molecules include dasatinib, a tyrosine kinase inhibitor first studied in the context of chronic myeloid leukemia (CML); quercetin, a dietary flavonoid found in onions, apples, and capers; and fisetin, another dietary flavonoid (present in strawberries, apples, persimmons, and various vegetables). Dasatinib is especially effective against senescent human adipose-derived progenitor cells and senescent endothelial cells, and Quercetin and Fisetin typically only target senescent endothelial, fibroblast, and some immune cell populations [22]. Interestingly, the mechanism of action of senomorphics differs from that of senolytics, as it modifies the senescent cell phenotype and decreases SASP expression without necessarily initiating apoptosis. Senomorphics may be beneficial for some tissues that do not benefit from the complete ablation of senescent cells. Mechanistically, Quercetin and Fisetin exhibit metal-dependent prooxidant activity, generating ROS in the presence of redox-active metals, such as copper and iron, which are enriched in senescent cells. This oxidative stress selectively damages senescent cells while leaving normal cells relatively unharmed [23]. Fisetin further modulates nuclear factor kappa-light-chain-enhancer of activated B cells (NF-κB) signaling, suppressing SASP factor expression in surviving senescent cells, thereby combining senolytic and senomorphic actions [22].

Senolytics have been shown to reduce the burden of senescent cells and promote healthspan in preclinical studies. In aged mice, intermittent doses of Dasatinib + Quercetin reduced the senescent cell count in multiple tissues and improved insulin sensitivity, cardiac function, and exercise endurance [24]. In models of Alzheimer’s disease, Fisetin treatment improved cognitive performance, reduced neuroinflammation, and outperformed Dasatinib + Quercetin in preserving function in some genotypes and sexes [25]. Fisetin also protects against age-related bone loss in progeroid mouse models by preserving osteoprogenitor pools and reducing osteoclastogenesis [26]. The clinical translation of senolytics is currently ongoing. A human pilot study involving patients with diabetic kidney disease (DKD) demonstrated that oral administration of Dasatinib + Quercetin as an intermittent treatment for three days resulted in a decrease in the burden of senescent cells in the adipose tissue and skin at three months, reduced SASP factors in circulation, and improved physical function (Table 1) [27]. Further early-phase trials are underway to investigate different indications of senolytic activity in idiopathic pulmonary fibrosis, osteoarthritis, frailty, and Alzheimer’s disease, with early data suggesting improvements in mobility, systemic inflammation, and quality of life [28]. However, there are also limitations to the translatability of findings from animal to human studies because the mechanisms of the immune system differ between species, and there are differences in the composition of tissues and drug metabolism, which may lead to different safety and efficacy profiles.

Several preclinical studies have employed relatively high AA dosing strategies, such as 1% AA in rodent chow, to investigate its impact on Alzheimer’s disease models. This concentration was selected because of its previously reported efficacy in modifying oxidative stress and mitochondrial function; however, it may represent supraphysiological exposure compared to human supplementation. Therefore, translational relevance must be considered in the context of pharmacokinetic data. In humans, plasma concentrations of AA rarely exceed 80–100 µM following high-dose oral administration because the AA absorption is saturable. In contrast, IO can transiently elevate plasma concentrations to the millimolar range. This highlights the promise and challenges of translating preclinical research into clinical settings. Another limitation of animal study data is that the plasma concentrations of AA often vary greatly, complicating the interpretation of dose–response relationships. Therefore, greater emphasis should be placed on this variability when considering AA as a gerotherapeutic agent. Sex-specific responses represent another dimension of this study. AA improvements in behavior were mostly reported in female mice, whereas mitochondrial and antioxidant improvements were observed in both sexes, albeit less pronounced in males. This should be noted as a limitation of the design because hormonal status likely alters both behavioral and metabolic outcomes in females. It will be important for future experimental designs to stage hormones to better correlate these findings. Finally, while several reports conclude that AA’s beneficial effects occur independently of Aβ plaque burden, this interpretation should be nuanced by considering soluble oligomeric Aβ species. These oligomers are now recognized as key drivers of synaptic dysfunction and cognitive decline, and it remains plausible that AA may influence such soluble species, even if the plaque load is unaffected [2,3,29,30].

**Table 1:** Comparative safety profiles and side effects of major anti-aging drug classes.

Drug Class	Common Side Effects	Long-Term Safety Concerns	Contraindications/Notes	Ref.
Metformin	Gastrointestinal upset, vitamin B12 deficiency	Rare lactic acidosis (in renal impairment)	Avoid in patients with severe kidney disease	[23]
Rapamycin/Rapalogs	Mouth ulcers, insulin resistance, hyperlipidemia	Immunosuppression, wound healing	Intermittent dosing may reduce risks	[31]
Senolytics (Dasatinib + Quercetin, Fisetin)	Fatigue, gastro-intestinal distress (Dasatinib + Quercetin); well-tolerated (Fisetin)	Potential off-target apoptosis, cytopenia	May require personalized or pulsed regimens	[27]
NAD^+^ precursors (NMN, NR)	Mild flushing, nausea	Theoretical tumor-promoting risk (ongoing debate)	Well-tolerated in trials up to 900 mg/day	[32]
Senomorphics (JAK inhibitors)	Headache, anemia, and infection risk	Long-term immunosuppression, possible malignancy risk	Monitor liver function, use in low doses if chronic	[33]

Abbreviations: NAD^+^, Nicotinamide Adenine Dinucleotide; NMN, Nicotinamide Mononucleotide; NR, Nicotinamide Riboside; JAK, Janus Kinase.

Notwithstanding these innovations, challenges remain to be addressed. The heterogeneity of senescent cells implies that there is no ideal senolytic approach for targeting these cells. The tissue- and stimulus-specific nature of senescence suggests that individualized or combination approaches will be needed. Off-target effects are a concern, such as transient cytopenias with Dasatinib or the possibility of hepatotoxicity with high-dose flavonoids, which require safety ascertainment. While it is reasonable to preferentially use intermittent administration to limit side effects while leaving time for senescent cell clearance, dosing schedules are still being formalized, and such assumptions should be tested in larger, longer trials [34,35,36,37,38].

In the future, senolytics have the potential to be a fundamental component of “gerotherapeutics” regimens when combined with senomorphics, NAD^+^ boosters (e.g., nicotinamide riboside), autophagy stimulators, or anti-inflammatory agents, which could lead not only to delaying the onset of age-related diseases but also to reversing some aspects of tissue dysfunction thought to be irreversible.

### 3.2. NAD^+^ Precursors: Nicotinamide Riboside (NR) and Nicotinamide Mononucleotide (NMN)

Nicotinamide adenine dinucleotide (NAD^+^), a pyridine nucleotide, serves two roles: (1) as a universal redox coenzyme in cellular metabolism and (2) as a substrate for enzymes that use NAD^+^ as a substrate, such as cyclic ADP-ribose synthases, CD38, and CD157 [32]. This list also includes the SIRT family of deacylases (PARPs). These NAD^+^-utilizing enzymes control many important areas of longevity biology, including proteostasis, mitochondrial biogenesis, circadian regulation of metabolism, genomic integrity, and inflammatory signaling [39]. Depletion of NAD^+^ levels with aging, which is observed in multiple tissues in mammals, is due in large part to decreased biosynthesis and increased NAD^+^ consumption, including inflammation, which initiates increased NADase consumption, including CD38 [40]. NAD^+^ depletion has many consequences, including loss of mitochondrial function, loss of stress response capacity, promotion of a state of “inflammaging” and depletion of stem cells. NAD^+^ depletion is not only a characteristic of aging but also of a myriad of diseases, including metabolic syndrome, neurodegenerative disease, cardiovascular disease, and acute kidney injury. This indicates the breadth of NAD^+^ usage and its role in mediating cellular resilience and energy homeostasis. Interestingly, there are NAD^+^ precursors, nicotinamide riboside (NR) and nicotinamide mononucleotide (NMN), which are ubiquitous intermediates in the NAD^+^ salvage pathway. NR is first phosphorylated to NMN by nicotinamide ribose kinases (NRKs), which are abundant in several tissues. This reaction is followed by NMN’s adenylation to NAD^+^ by NMN adenylyltransferases (NMNATs), which are abundantly expressed in several tissues. NMN can enter cells via two mechanisms. NMN can either enter the cell as NR or remain intact, as with previously identified NMN-specific transporters, such as SLC12A8 (a newly described NMN importer expressed in murine enterocytes) [39,41]. Transporters are regulated depending on the tissue, potentially leading to significant differences in the distribution and response to various NAD^+^ precursors. Recent evidence suggests that the extent to which they are converted to nicotinamide (NAM) in extracellular circulation before their return to the salvage pathway could be more dynamic than previously assumed. More research is needed to clarify the potential and distinction between local and systemic NAD^+^ metabolism.

Supplementation with NMN and NR enhances tissue NAD^+^ levels and triggers downstream sirtuin-dependent pathways, according to preclinical data from murine models. NMN improves endothelial vasodilation, insulin sensitivity, mitochondrial biogenesis, and oxidative phosphorylation in elderly rats by deacetylating endothelial nitric oxide synthase (eNOS) via SIRT1 [42]. NMN also improves muscle oxidative metabolism and endurance and lowers age-related weight gain by improving mitochondrial quality control and decreasing inflammatory signals [41]. Similar outcomes have been demonstrated with NR, which has been proven to improve heart function following myocardial infarction, reverse cognitive decline in Alzheimer’s disease mouse models, and prevent noise-induced hearing loss [43]. Notably, NR and NMN may differ in tissue targeting, with NR showing stronger effects in the brain and muscles and NMN appearing more effective in the liver and vascular tissues. Human trials with NR and NMN are still in their early stages, but they have established strong safety and pharmacokinetic profiles. Multiple randomized, placebo-controlled studies have shown that oral NR at dosages up to 2 g/day and NMN at levels up to 900 mg/day are well tolerated, with no significant side effects [44,45]. Supplementing healthy middle-aged and older individuals has been shown to enhance circulating NAD^+^ metabolites and lower systolic blood pressure and arterial stiffness [46]. Over 10 weeks, NMN administration in postmenopausal women enhanced insulin sensitivity and muscle remodeling gene expression without changing body composition [47]. Despite considerable increases in NAD^+^ metabolite levels, the clinical outcomes have been variable. Some trials demonstrated minimal physiological benefits, probably due to variability in population health status, baseline NAD+ levels, trial duration, and the inadequate power to detect minor effects. Furthermore, there is growing concern regarding the possibility that NAD^+^ repletion could promote cancer progression. Elevated NAD^+^ levels improve oxidative metabolism, DNA repair, and stress tolerance, which may increase the survival of tumor cells. Although preclinical research has yielded inconsistent findings based on tumor type and mutation status, caution should be exercised when considering long-term supplementation in high-risk individuals [32,48]. Another drawback is the absence of direct human trials comparing NR and NMN in terms of pharmacokinetics, effectiveness, and tissue distribution. Individual genetic variations, transporter expression, and disease-specific targets may ultimately determine the best precursor for a given condition.

Future trials, including those on NMN in frail older persons and NR in metabolic syndrome, will determine whether NAD^+^ precursor supplementation can extend the human healthspan. Combining NAD^+^ precursors with CD38 inhibitors, sirtuin activators, or mitochondrial-targeted antioxidants can improve efficacy and reduce the requirement for high doses [49]. Next-generation NAD^+^ precursors, including dihydronicotinamide riboside (NRH) and reduced NMN (NMNH), are being developed to improve bioavailability by bypassing the rate-limiting steps in the salvage pathway.

### 3.3. mTOR Inhibitors: Rapamycin and Rapalogs

mTOR is a serine/threonine kinase that acts as a hub for food, energy, and growth factor sensing, integrating these signals to control cell growth, protein and lipid synthesis, mitochondrial biogenesis, and autophagy [50]. mTOR functions in two different complexes: mTORC1, which is initially responsive to the macrolide rapamycin, and mTORC2, which is less sensitive but can be blocked by persistent rapamycin treatment. Chronic mTORC1 overactivation is a hallmark of aging, promoting anabolic processes at the expense of cellular maintenance, inhibiting autophagy, weakening stress resistance, and hastening the onset of age-related diseases [51]. In addition to aging, aberrant mTOR signaling contributes to a broad spectrum of chronic diseases, including type 2 diabetes, neurodegenerative disorders, cardiovascular diseases, and certain cancers. mTOR’s dual role in supporting cell growth and inhibiting stress adaptation makes it a key target in both geroscience and oncology.

The most effective pharmacological agent for extending longevity across species is rapamycin, which was first isolated from *Streptomyces hygroscopicus* on Easter Island. Its effects have been demonstrated in both yeast and human studies. Even when treatment is initiated late in life, rapamycin increases both median and maximal lifespans in genetically diverse mice, indicating that its geroprotective mechanisms are independent of early life exposure [52]. The advantages go beyond longevity; in mouse models, rapamycin prevents cognitive impairment, maintains diastolic cardiac function, delays the onset of neoplasia, and improves age-related immune function losses [53]. Chronic rapamycin administration has been well tolerated in primates, reducing mTORC1 signaling, maintaining renal function, and reaching clinically relevant drug concentrations without causing notable immunological or metabolic abnormalities [54]. Without causing significant side effects, it has been demonstrated that short-term, low-dose treatment of rapamycin or rapalogs like everolimus can reverse some features of immunosenescence in humans and improve the antibody response to influenza vaccination in older people [31]. Although there may be modest mucocutaneous and gastrointestinal side effects, pilot studies in older adults have shown that rapamycin can be administered safely for weeks to months without affecting glucose tolerance, physical function or cognition [55]. A noteworthy pharmacological precedent for their possible repurpose as gerotherapeutics is the FDA approval of rapalogs for various purposes, including organ transplantation and oncology.

The principal anti-aging mechanism of rapamycin is mTORC1 suppression, which, like calorie restriction, promotes a metabolic state that supports cellular repair and maintenance by suppressing S6K1 and 4E-BP1 activity, lowering cap-dependent translation, and activating autophagy [56]. Conversely, long-term treatment may inadvertently suppress mTORC2, which lowers AKT phosphorylation and causes related issues with glucose and lipid balances [57]. This has increased interest in analogs such as DL001, which, in preclinical trials, displayed significantly better selectivity for mTORC1 while maintaining geroprotective effectiveness without causing mTORC2-related metabolic side effects [58]. In addition to DL001, several novel substances that engage both mTORC1 binding domains with less off-target toxicity, such as RapaLink-1 and bi-steric mTOR inhibitors, have improved pharmacodynamic profiles. Although these drugs are still in the early stages of development, they could eventually offer more precise treatment [59]. 

One of the main objectives of translational research is to reduce the negative effects of rapamycin while maintaining its benefits. Administering intermittent doses to mice, such as once every five days, increases lifespan in a manner comparable to continuous dosage while reducing the effects on immunological and glucose metabolism [60]. In both dogs and rats, short-course therapy starting in mid-to-late life has resulted in long-lasting functional benefits without chronic metabolic damage, even though rapalogs such as everolimus and temsirolimus have unique pharmacokinetic properties that lessen mTORC2 interference [54]. The optimal time, dosage, and duration of rapamycin administration in humans remain unclear, particularly when comparing healthy individuals with those suffering from age-related diseases. As shown in animal models, sex-specific variations in response must be carefully considered when planning human studies [61]. Drugs that combine mTOR inhibitors with metformin, NAD^+^ precursors, or senolytics that activate AMPK, promote autophagy, or restore insulin sensitivity are being investigated to maximize therapeutic advantages and reduce side effects [62].

### 3.4. Metformin: AMPK Activation and Longevity Potential

Metformin, the primary medication for type 2 diabetes for over 60 years, derives from Galega officinalis. The primary mechanism is the change in the AMP/ATP ratio, resulting in the activation of AMPK, which regulates the cellular energy balance. AMPK activation blocks metabolic pathways, such as protein translation and lipid synthesis, and activates catabolic pathways, including fatty acid oxidation and glucose uptake [63,64]. Metformin indirectly inhibits the mTORC1 pathway by activating the tuberous sclerosis complex 2 (TSC2) and Raptor. Multiple upstream pathways regulate autophagy and proteostasis in a manner analogous to rapamycin and caloric restriction [63]. AMPK activation also boosts mitophagy and the formation of new mitochondria via peroxisome proliferator-activated receptor gamma coactivator 1-alpha (PGC-1α) and Unc-51-like kinase 1 (ULK1), aiding in the maintenance of healthy mitochondria, which is a key part of aging. In addition to AMPK and mTOR changes, metformin reduces mitochondrial ROS levels by inhibiting complex I in the electron transport chain. This reduces oxidative stress, increases mitochondrial activity, and strengthens the cellular response to stress. All of these alterations address several fundamental signs of aging, including impaired nutrition sensing, mitochondrial dysfunction, and unstable genes. In addition, metformin lessens body-wide inflammation by reducing NF-κB activity and decreasing pro-inflammatory messengers in the blood [23]. Metformin also alters the gut microbiome, increases gut mucus production, and strengthens the gut barrier, which may affect body-wide inflammation and the balance of body metabolism.

Metformin may offer broad protection against a range of age-related diseases in humans. Compared to diabetic patients taking other drugs and, in certain studies, non-diabetic controls, metformin-treated T2DM patients exhibited decreased incidence rates of cancer, cardiovascular disease, cognitive impairment, and all-cause death in large retrospective cohorts [65,66]. Although these results are susceptible to confounding factors, they are corroborated by molecular discoveries and smaller interventional studies in people without diabetes, which showed that metformin enhances vascular function, metabolic flexibility, and inflammatory profile [63].

Emerging evidence suggests that metformin may improve immunological resilience in older adults by renewing hematopoietic stem cell activity and T-cell memory responses, although human studies are limited. Preclinical studies have indicated that metformin enhances longevity and slows functional decline in various model species, including *C. elegans*, *Drosophila melanogaster*, and numerous rat strains. However, its effects in mice differ depending on the strain and dosage of the drug [63]. The necessity of dosage control is highlighted by the possibility that large doses might paradoxically shorten rat lifespan through gastrointestinal damage or extreme energy stress. These benefits appear to be due to a combination of metabolic reprogramming, autophagy activation, and suppression of chronic low-grade inflammation at the recommended dose.

Metformin provides long-term advantages for preventing age-related disorders, extending the therapeutic window for intervention, in contrast to NAD^+^ precursors or rapamycin. The translational potential of metformin as a geroprotective agent is currently being tested in the Targeting Aging with Metformin (TAME) trial, a landmark, randomized, placebo-controlled, multicenter study designed to determine whether metformin can delay the onset of a composite endpoint of major age-related diseases, such as cancer, cardiovascular events, dementia, and mortality (Table 1). Beyond its clinical goals, TAME has significant regulatory implications. If successful, it may set a precedent for the formal recognition of aging as a modifiable and treatable condition, paving the way for the approval of future interventions targeting the biological aging process [67]. Furthermore, the TAME trial may serve as a template for evaluating other candidate gerotherapeutics, encouraging the use of composite endpoints that reflect the multifactorial nature of aging rather than focusing on single-disease outcomes.

### 3.5. Senomorphics and Anti-Inflammatory Agents

Cellular senescence is a stress-induced state of stable cell cycle arrest, accompanied by profound changes in chromatin organization, metabolism, and secretory activity. A hallmark of senescent cells is the SASP, a pro-inflammatory and tissue-remodeling secretome composed of cytokines, chemokines, growth factors, and proteases that can drive chronic inflammation, disrupt tissue homeostasis, and promote tumorigenesis. While senolytics aim to selectively eliminate senescent cells, senomorphics suppress or reprogram the SASP without inducing cell death, thereby mitigating deleterious paracrine effects while preserving the beneficial, context-dependent functions of senescent cells, such as tissue repair, wound healing, and tumor suppression [68]. This approach may be especially valuable in tissues where the clearance of senescent cells risks impairing structural integrity or regeneration, such as the skin, lungs, and cardiovascular system.

Senomorphic activity has been reported for several agents already recognized for their geroprotective effects through other mechanisms. Rapamycin and its analogs inhibit mTORC1, which directly affects SASP translation by suppressing IL-1α-mediated NF-κB activation [36]. Metformin lowers pro-inflammatory SASP factors such as IL-6, IL-8, and monocyte chemoattractant protein-1 (MCP-1) by activating AMPK and inhibiting NF-κB [23]. These drugs also promote autophagy, a process that aids in the destruction of inflammasome components and SASP regulators, thereby enhancing their senomorphic effects. JAK inhibitors are a more direct senomorphic strategy because JAK/STAT signaling is a critical upstream regulator of *SASP* gene transcription. When administered systemically to older mice, the JAK1/2 inhibitor ruxolitinib decreased SASP factors in many organs, improved hematological function, and decreased frailty [33]. Crucially, JAK inhibition may restore immune surveillance and potentially reduce inflammation that promotes tumors by slowing the age-related proliferation of myeloid-derived suppressor cells (MDSCs). Pharmacological suppression of pro-inflammatory pathways, such as JAK/STAT modulation with ruxolitinib or IL-6 signaling blockade with tocilizumab, is effective in lowering inflammatory biomarkers and improving disease phenotypes in age-related conditions, although long-term safety and infection risk remain important factors [69]. Further research is needed to determine whether these interventions can be safely implemented for chronic preventive use in aging populations without compromising host defense or immune resilience.

Repurposed agents also hold promise as senomorphic and anti-inflammatory agents. Nonsteroidal anti-inflammatory drugs (NSAIDs) have been associated with a reduced incidence of Alzheimer’s disease and certain cancers in observational studies, potentially via cyclooxygenase (COX) inhibition and downstream prostaglandin suppression. However, randomized controlled trials have produced mixed results, and concerns about gastrointestinal and cardiovascular toxicity persist. Natural compounds, such as resveratrol, curcumin, and β-caryophyllene, have been shown to attenuate SASP production and reduce inflammatory signaling in endothelial and immune cells in vitro [70]. However, poor bioavailability and off-target effects limit their translational potential unless they are optimized through formulation or delivery technologies. Emerging research has identified the gut microbiome as a pivotal modulator of systemic inflammation and immune function during aging. Age-associated dysbiosis can promote SASP induction through increased intestinal permeability and microbial metabolite imbalance, leading to endotoxemia and chronic immune activation in the elderly. Interventions that restore a eubiotic microbiome through dietary fiber, prebiotics, probiotics, or fecal microbiota transplantation have demonstrated reductions in systemic inflammatory markers and improvements in metabolic health in preclinical and early human studies [36]. Microbiota-mediated modulation of tryptophan metabolism, short-chain fatty acid production, and bile acid signaling has also been implicated in the regulation of SASP activity and tissue inflammation. Precision senomorphics, such as aptamer-conjugated compounds that specifically alter SASP factor release in certain cell types to prevent widespread immunosuppression, have also been investigated recently [71]. By modifying rather than completely blocking senescence-associated signals, this strategy seeks to strike a balance between the physiological benefits and drawbacks of the senescent state. Other intriguing approaches include siRNAs targeting SASP regulators delivered via nanoparticles and context-specific small molecules designed to inhibit only the inflammatory or fibrotic components of the SASP without impairing regenerative signals. Ultimately, senomorphics offer a flexible, tissue-sparing strategy for mitigating the negative effects of senescence and may be particularly useful in combination therapies with senolytics, NAD^+^ precursors, or mTOR inhibitors to optimize the healthspan while minimizing toxicity.

### 3.6. SGLT2 Inhibitors

Sodium-glucose co-transporter-2 inhibitors (SGLT2i), which were originally authorized for antihyperglycaemic purposes in the treatment of type 2 diabetes mellitus (T2DM), have recently been described as geroprotective agents owing to their pleiotropic pathways that affect some hallmarks of aging, including insulin resistance, oxidative stress, mitochondrial dysfunction, and systemic inflammation. Moreover, in addition to their established cardioprotective and renoprotective effects, the possibility of SGLT2i to elongate healthspan via their effects on metabolism and broad cellular pathways underlying the biology of aging has also been reported [72]. SGLT2i improves insulin sensitivity by alleviating glucotoxicity and hyperinsulinemia via urinary glucose loss, which further benefits adipose tissue function, lipid metabolism, and overall systemic nutrient sensing. Moreover, SGLT2i have a strong antioxidative effect, supporting both animal and human studies describing reductions in reactive oxygen species (ROS) with improvements in both mitochondrial biogenesis and oxidative phosphorylation, and reductions in NLRP3 inflammasome activation, which all support a reduction in oxidative stress and chronic low-grade inflammation. Improving endothelial function and nitric oxide bioavailability are important contributors to their vascular protective effects. Further, there is emerging evidence to suggest potential neuroprotective actions, including the reduction in neuroinflammation, improvement in synaptic plasticity, and improvement in neurotrophic factor signaling, which may be relevant to age-associated cognitive decline [73,74,75,76,77,78,79,80].

Numerous clinical studies have documented the multidimensional benefits in geriatric populations. In older patients diagnosed with T2DM and heart failure with preserved ejection fraction (HFpEF), SGLT2i therapy was linked to changes in cognitive and depressive aspects, and showed beneficial changes in biomarkers of oxidative stress and platelet activation [81]. Likewise, in older patients hospitalized for heart failure with reduced ejection fraction (HFrEF), the everyday use of SGLT2i led to significantly greater improvements in Mini-Mental State Examination (MMSE) scores than those who were not treated with SGLT2i, consistent with a direct association with cognitive trajectories [82]. A recent longitudinal study showed that the combination of SGLT2i and sacubitril/valsartan resulted in enhanced echocardiographic measures, decreased oxidative stress biomarkers, and increased muscle and cognitive function, suggesting complementary benefits in geriatric heart failure populations [83]. Armentaro’s work agrees with experimental data indicating a decrease in mitochondrial ROS production with SGLT2i treatment and improvements in frailty indices in older patients treated with SGLT2i.

Support for these claims comes from meta-analyses conducted by other researchers. One meta-analysis including over 2200 patients reported significant reductions in insulin resistance measures (HOMA-IR) associated with SGLT2i compared to placebo, demonstrating their metabolism-sustaining relevance with age [84]. A systematic review also confirmed that SGLT2i consistently reduced oxidative stress biomarkers, thereby validating their role as systemic antioxidants [85]. Ultimately, large meta-analyses of heart failure populations showed improvements in overall peak VO_2_, six-minute walk distance, and quality of life measures for both diabetic and non-diabetic individuals. Overall, gains in physical capacity translate directly to reduced frailty and greater independence among older adults [86].

These data suggest the potential of SGLT2i as therapeutic candidates for geriatric practice. By simultaneously targeting insulin resistance, oxidative stress, inflammation, and functional status, they act on many hallmarks of aging, as they have clinically relevant effects on cognition, physical function, and frailty. Studies to date have limitations with short follow-up periods, small study sizes, varying patient populations, and low-level functional and cognitive measures. Studies that follow older persons who are not clinically diabetic in longer randomized controlled trials while using a sensitive biomarker of biological age and neurocognitive testing to determine if SGLT2i could actually delay or reverse the timelines of aging beyond disease-specific benefits will be needed. It may ultimately contribute to the current geroprotective evaluation of therapeutics, alongside metformin, NAD^+^ precursors, and mTOR inhibitors, and increase the therapeutic options for pharmacological agents to target the longevity healthspan.

## 4. Comparative Analysis of Drug Classes

Senolytics, NAD^+^ precursors, mTOR inhibitors, biguanides such as metformin, and senomorphics are the major categories into which new pharmacological approaches for human anti-aging therapy can be classified (Table 2). Although these drugs have the same overall objective of prolonging healthspan, they vary greatly in their mechanistic scope, translational maturity, safety profiles, and potential for synergistic application. Prioritizing candidates for clinical deployment requires critical comparative analysis of various pharmacological agents. In evaluating these agents, it is crucial to consider their alignment with the nine hallmarks of aging, as this offers a framework for systematic comparison and rational combination therapies. Mechanistic breadth is a defining parameter for assessing anti-aging drug classes. By specifically inducing apoptosis in senescent cells, senolytics such as Dasatinib, Quercetin, and fisetin exhibit high specificity, directly addressing the hallmarks of cellular senescence and indirectly reducing “inflammaging” by lowering the SASP [87]. However, they have little effect on other characteristics such as telomere attrition and genomic instability. NAD^+^ precursors, NMN and NR, have various functions, including DNA repair, mitochondrial function, sirtuin activity, and stem cell maintenance [42,88]. mTOR inhibitors, such as rapamycin and its analogs, impact autophagy, proteostasis, and tissue regeneration, despite their primary target being dysregulated nutrition sensing [89,90]. Metformin, though mechanistically narrower than NAD^+^ boosters or mTOR inhibitors, exerts multi-hallmark effects through AMPK activation, mitochondrial efficiency enhancement, and reduction in oxidative stress [63,65]. Senomorphics, including rapamycin in its SASP-modulatory capacity and JAK/STAT pathway inhibitors, offer a subtler approach by suppressing the deleterious effects of senescent cells without eliminating them, which may preserve beneficial senescence-related functions such as wound repair [91]. Notably, some agents, such as rapamycin and metformin, exert overlapping effects on nutrient sensing, autophagy, and inflammation, highlighting the potential for additive or synergistic combinations.

From a translational perspective, the maturity of each drug class varies significantly among them. With decades of clinical use and epidemiological evidence indicating lower rates of cardiovascular events, cancer incidence, and all-cause mortality in diabetes cohorts, metformin is at the forefront of diabetes treatment [65]. Owing to their well-established use in oncology and transplantation, mTOR inhibitors also benefit from a wealth of human pharmacokinetic and safety data. However, their immunosuppressive potential and metabolic side effects require careful dose optimization and possibly intermittent regimens [92]. NAD^+^ precursors have advanced to early-phase clinical trials, demonstrating safety, tolerability, and improvements in surrogate markers such as vascular function and NAD^+^ levels. However, long-term outcome data are lacking [46]. Senolytics have shown robust lifespan and healthspan extensions in multiple animal models [93,94]. Current human evidence is confined to small-scale studies demonstrating reductions in the senescent cell burden and improvements in physical function [95]. Senomorphics remain largely experimental, with potential applications in chronic, low-toxicity regimens aimed at attenuating systemic inflammation in the long term. The development of more selective, cell-type–specific senomorphic agents is actively underway and may improve safety and efficacy in future clinical applications.

In the field of preventive pharmacotherapy for aging, safety is of utmost importance, especially when it comes to therapies intended for use in otherwise healthy older individuals. Despite the promising nature of senolytics, intermittent doses may alleviate concerns regarding off-target apoptosis and the possible impairment of tissue repair mechanisms [18]. Pulsed delivery or targeted mTORC1 targeting can mitigate the effects of chronic mTOR suppression, which are linked to glucose intolerance, dyslipidemia, and poor wound healing [96]. Although concerns have been expressed over the potential risk of increasing tumor metabolism in some situations, NAD^+^ precursors are generally well tolerated in the short term, requiring careful long-term monitoring [97]. Metformin has an exceptional safety record but is contraindicated in individuals with significant renal impairment due to the rare risk of lactic acidosis. Senomorphics, such as JAK inhibitors, carry infection and malignancy risks when used chronically at higher doses [98]. Moreover, the safety profile of each drug may be modulated by patient-specific factors, such as age, frailty, comorbidities, and polypharmacy, reinforcing the need for personalized risk stratification in gerotherapeutic trials.

Given the interconnectedness of the hallmarks of aging, combination or sequential therapeutic approaches may ultimately prove superior to monotherapy. For example, intermittent senolytic “debulking” to clear accumulated senescent cells could be followed by NAD^+^ precursor supplementation to optimize mitochondrial recovery and stem cell function. Similarly, the co-administration of metformin and low-dose rapamycin has been hypothesized to synergistically modulate nutrient-sensing pathways while minimizing mTOR-related metabolic liabilities [61]. Emerging studies suggest that multi-drug regimens targeting complementary hallmarks may produce additive or even synergistic gains in healthspan, especially if guided by biomarkers of biological aging, such as DNA methylation clocks, SASP factor panels or circulating NAD^+^ levels. The design of such regimens will require careful balancing of mechanistic complementarity with toxicity management, a challenge best addressed through adaptive biomarker-driven clinical trials.

Finally, economic and regulatory considerations will heavily influence the clinical integration of these interventions in the future. Low-cost, widely available drugs, such as metformin, have the greatest potential for rapid, broad deployment if their efficacy is confirmed, whereas expensive, proprietary molecules may initially be restricted to high-risk or affluent populations. The absence of regulatory recognition of aging as a treatable indication remains a fundamental bottleneck. However, if pivotal studies, such as TAME or rapamycin vaccination-enhancement trials, succeed, they may catalyze a paradigm shift in drug development, reimbursement, and clinical practice, with profound implications for public health and healthcare economics. In the long term, regulatory frameworks may evolve to support composite aging endpoints, companion diagnostics, and life-course interventions, thereby reshaping the approach to chronic disease prevention and healthy aging.

To provide an overview of pharmacological strategies targeting the hallmarks of aging, we compiled a comparative table summarizing key drug classes, their primary mechanisms, and evidence in aging and age-related disease models (Table 1). Importantly, recent findings on SGLT2 inhibitors, which were not initially included, have been added here, given their emerging role in modulating oxidative stress, insulin resistance, and functional outcomes in elderly patients.

## 5. Current Clinical Trials and Evidence

With an expanding portfolio of early- and late-phase clinical studies, the translation of anti-aging pharmacological methods from preclinical models to humans has advanced significantly over the past decade. Based on the idea that addressing common characteristics may concurrently postpone or prevent several chronic diseases, this changing clinical landscape represents a move away from disease-specific symptom management toward the upstream control of biological aging. Owing to the vast retrospective epidemiological evidence and current prospective evaluation in the TAME trial, metformin continues to be the most advanced medication in terms of its clinical translation. To determine whether metformin can delay the onset of age-related multimorbidity, TAME is a multicenter, randomized, placebo-controlled study with composite endpoints encompassing mortality, cardiovascular disease, cancer, and cognitive decline (Table 3) [67].

Rapamycin and its analogs (rapalogs) are being evaluated in human aging-related contexts, particularly immunosenescence. Influenza vaccine responses in older adults were improved by short-term rapalog everolimus in a randomized trial, which also showed a decrease in circulating pro-inflammatory cytokines [92]. The capacity of rapamycin to enhance immunological resilience, heart function, and skin aging characteristics in older persons is still being studied, and further research has improved the dosing schedules to reduce negative metabolic consequences. A possible tactic to maintain effectiveness while reducing mTORC2 inhibition and related metabolic risks is intermittent or pulsed dosing. NAD^+^ precursors have rapidly transitioned from preclinical validation to early-phase human trials. NR supplementation has been evaluated in randomized controlled settings, demonstrating significant increases in circulating NAD^+^ levels, alongside modest improvements in blood pressure and arterial stiffness in middle-aged and older adults [46]. NMN has similar NAD^+^-boosting effects in humans, with a recent placebo-controlled trial reporting improved insulin sensitivity in postmenopausal women with prediabetes [47]. These trials confirm bioavailability and metabolic engagement. However, the effects on clinical endpoints such as frailty, cognitive function, or physical performance remain to be demonstrated. Larger, longer-duration trials are required to assess the durability and long-term safety of this treatment. Senolytics are in the early stages of human translation, with most evidence derived from pilot studies targeting certain age-related illnesses. In an open-label experiment, a combination of Dasatinib and Quercetin, administered to patients with idiopathic pulmonary fibrosis, improved physical function tests and reduced markers of senescent cell load in peripheral organs [95]. Another short experiment on DKD showed mild functional benefits and decreased senescence biomarker levels [27]. Preliminary findings suggest that Fisetin, when used alone as a senolytic, has good tolerability and may be effective in reducing frailty traits. Although conceptually attractive for chronic low-dose administration, senomorphics remain underrepresented in aging-focused clinical research. However, indirect evidence from trials of JAK inhibitors in inflammatory and fibrotic conditions supports their potential to modulate systemic inflammation in age-associated diseases. Large preventive trials have also examined the effects of low-dose aspirin and other anti-inflammatory drugs. However, their inconsistent effectiveness and bleeding risks underscore the need for more focused approaches [99]. The repurposing of anti-cytokine medicines, such as IL-6 or TNF-α inhibitors, for aging applications is gaining traction, especially in disorders such as cognitive decline, sarcopenia, and frailty.

The use of disease-specific or surrogate endpoints instead of proven indicators of biological age or healthspan is a prevalent restriction in the current clinical environment. Standardized biomarkers, such as proteomic signatures, multi-omic aging indices, or epigenetic clocks, are urgently needed to standardize trial results and facilitate cross-study comparisons, even though composite endpoints, such as those in TAME, are a step in the right direction toward capturing the systemic aspect of aging [100]. In addition, most studies have relatively short follow-up periods and involve individuals with few age-related diseases, which restricts their ability to detect long-term improvement. Enhancing signal detection and expediting regulatory approval may be achieved using enrichment approaches to target high-risk categories, such as older individuals with metabolic syndrome, frailty, or multimorbidity. To assess combinations, modify dosages, and tailor therapies according to biological age or molecular phenotype, novel trial designs such as adaptive platform trials, N-of-1 designs, and longitudinal multi-arm cohorts are being investigated.

Previously considered speculative interventions, therapeutic tactics such as systemic increase in NAD^+^ levels and the removal of senescent cells are now partially validated by empirical data from human trials. Whether pharmacological methods to control the aging process move from exploratory research to routine medical use will depend on the direction of these studies over the next ten years, as well as developments in biomarker development and the creation of suitable regulatory guidelines. Ultimately, the development of easily applicable biomarkers, validation of significant clinical benefits, and implementation of uniform regulatory requirements are necessary for the broad usage and safety profile of these pharmacological agents. The first generation of medications that can postpone the onset of certain chronic diseases may soon be available, thanks to advances in genomics.

## 6. Conclusions

Pharmacological modulation of human aging has advanced from theoretical speculation to an active area of translational science. However, substantial gaps remain in the evidence base, mechanistic understanding, and regulatory pathways required for clinical integration. Future research must address these deficiencies through coordinated multidisciplinary efforts that bridge molecular biology, clinical trial design, biomarker development and public health policy. As gerotherapeutics approach clinical viability, the field faces a dual imperative: to deepen mechanistic insight while building scalable, safe, and equitable pathways for clinical deployment. The development of biological age indicators that are both responsive to pharmacological interventions and indicative of significant therapeutic effects is a top priority. Current approaches, such as DNA methylation-based epigenetic clocks [100,101], proteomic aging indicators, and composite physiological indices, lack universal standardization and validation across various populations, despite their promising platforms. To develop surrogate markers that can accelerate early-phase drug evaluation and promote cross-trial comparability, longitudinal studies combining multi-omics profiling with functional goals are required. Deep learning-based face and voice age predictions, metabolomic aging signatures, extracellular vesicle content, and immune cell phenotyping are novel biomarkers that may enhance sensitivity to short-term intervention effects and supplement conventional methods.

Another pressing research gap concerns the optimization of dosing strategies for gerotherapeutics, particularly agents such as rapamycin and senolytics, where the therapeutic window is narrow and chronic toxicity remains a concern. Adaptive trial designs that incorporate real-time biomarker feedback could help identify regimens that balance efficacy and safety, especially in preventive contexts where risk tolerance is low. In parallel, exploration of combinatorial or sequential interventions, such as intermittent senolytic therapy followed by chronic metabolic modulation with NAD^+^ precursors or metformin, may yield additive or synergistic benefits by addressing multiple aging hallmarks in a temporally coordinated manner. Computational modeling and systems biology approaches could assist in optimizing these multi-agent regimens by simulating hallmark-level responses and predicting potential adverse interactions before clinical testing.

The field must also confront the heterogeneity of aging trajectories across individuals. Genetic, epigenetic, environmental, and socioeconomic factors shape the onset and progression of age-related decline, implying that personalized or stratified approaches are necessary to maximize benefits. Research integrating polygenic risk scores, metabolic profiling, and lifestyle factors into trial recruitment and treatment assignment could reveal the population subgroups most likely to respond to specific pharmacological strategies. Artificial intelligence and machine learning techniques may enable more dynamic stratification and adaptive dosing algorithms, tailoring interventions in real time based on individual biological age and treatment response.

Furthermore, the unforeseen consequences and long-term safety of altering fundamental human aging pathways remain unknown. Long-term alterations in processes such as mTOR inhibition, senescence clearance, and NAD^+^ augmentation can lead to immunological dysregulation, tumor promotion in sensitive tissues, and poor wound healing. While short-term research indicates that many drugs are tolerable, registry-based follow-up and specialized post-marketing surveillance will be critical after the widespread adaption of these treatments are widely adopted. Better aged-animal models, human organoids, and in silico methods are needed to forecast long-term dangers, as preclinical models frequently miss complicated late-life comorbid relationships.

From a translational perspective, expanding the diversity of clinical trial populations is critical. The generalizability of many recent studies is hindered by the disproportionate use of wealthy, healthy participants from high-income countries. To promote equity and adequately reflect the biology of aging, individuals from diverse socioeconomic, racial, and comorbidity backgrounds should be included. International partnerships, including multi-site trial networks in low- and middle-income nations, could increase demographic representation and expand the body of information for a wider range of people.

Finally, the regulatory and economic landscapes represent major research and implementation gaps. The absence of a recognized clinical indication for “aging” remains a barrier to large-scale trials and pharmaceutical investments. Ongoing efforts by advocacy groups, academic consortia, and trialists, such as those surrounding the TAME initiative, must be expanded into a global framework for defining, measuring, and approving interventions that target aging itself.

As has been explored in oncology and rare diseases, there is increasing agreement that conditional regulatory approval for anti-aging therapies may be made possible by the establishment of surrogate endpoints, such as validated biological age reduction or delay in the development of multi-morbidity. To make a case for policy-level adoption, parallel economic evaluations are required to model healthcare savings and the societal advantages of extending healthspan.

## Figures and Tables

**Table 2:** Comparative analysis of drug classes.

Drug Class	Primary Mechanism(s)	Evidence in Aging/Age-Related Disease Models	Ref.
Metformin	AMPK activation, inhibition of mTOR, reduction in ROS, and improved insulin sensitivity	Extends lifespan in multiple species; reduces cancer risk, frailty markers, and cardiovascular events in humans	[23,63,65,67]
Rapamycin/mTOR inhibitors	Direct inhibition of mTORC1 modulates autophagy and proteostasis	Extends lifespan in yeast, worms, flies, and mice; delays age-related decline	[31,50,52,53,54,92]
NAD^+^ precursors (NMN, NR)	Boost NAD^+^ levels; activate sirtuins and PARPs; enhance mitochondrial function	Improve metabolism, DNA repair, and vascular function in preclinical models; early human data suggest improved muscle and cognitive function	[32,39,41,44,45,46,47,88]
Senolytics (dasatinib, quercetin, fisetin, etc.)	Selective elimination of senescent cells; reduce SASP factors	Improve physical function, delay frailty, and extend healthspan in mice; early clinical trials ongoing	[18,22,24,25,26,27,28,93,94,95]
SGLT2 inhibitors (empagliflozin, dapagliflozin, etc.)	Lower glucotoxicity, improve insulin sensitivity, reduce ROS and NLRP3 inflammasome activation, improve endothelial and mitochondrial function	Improve survival, reduce heart failure hospitalizations, preserve renal function; recent studies show improved cognition, mood, and functional outcomes in elderly HF patients	[72,73,74,75,76,77,78,79,80,81,82,83,84,85,86]

**Table 3:** Comparative clinical trial landscape of anti-aging agents.

Drug Class	Compound	Trial Name/ID	Target Population	Primary Endpoint	Ref.
Metformin	Metformin	TAME trial	Older adults (non-diabetic)	Delay of age-related diseases	[67]
Senolytics	Dasatinib + Quercetin	NCT02848131	Diabetic kidney disease	Senescent cell markers, physical function	[46]
NAD^+^ precursors	Nicotinamide, riboside	NCT02678611	Older adults	Arterial stiffness, NAD^+^ levels	[46]
NAD^+^ precursors	Nicotinamide, mononucleotide	NCT03151239	Postmenopausal women	Insulin sensitivity, muscle gene expression	[47]
mTOR inhibitors	Everolimus	NCT01190620	Elderly (65+)	Vaccine response, cytokines	[92]

Abbreviations: NAD^+^, Nicotinamide Adenine Dinucleotide; mTOR, Mechanistic Target of Rapamycin.

## Abbreviations

The following abbreviations are used in this manuscript:

AMPK	AMP-Activated Protein Kinase
ATP	Adenosine Triphosphate
BCL-2	B-Cell Lymphoma 2
CML	Chronic Myeloid Leukemia
CD38	Cluster of Differentiation 38
COX	Cyclooxygenase
DALY	Disability-Adjusted Life Year
DKD	Diabetic Kidney Disease
DNMT	DNA Methyltransferase
EPO	Erythropoietin
eNOS	Endothelial Nitric Oxide Synthase
FDA	Food and Drug Administration
G-CSF	Granulocyte Colony-Stimulating Factor
HALE	Healthy Life Expectancy
HDAC	Histone Deacetylase
HSP	Heat Shock Protein
IL	Interleukin
JAK	Janus Kinase
MDSC	Myeloid-Derived Suppressor Cell
MMP	Matrix Metalloproteinase
mTOR	Mechanistic Target of Rapamycin
mTORC1	Mechanistic Target of Rapamycin Complex 1
mTORC2	Mechanistic Target of Rapamycin Complex 2
NAM	Nicotinamide
NAD^+^	Nicotinamide Adenine Dinucleotide
NADH	Nicotinamide Adenine Dinucleotide (Reduced Form)
NADase	Nicotinamide Adenine Dinucleotide Hydrolase
NAMPT	Nicotinamide Phosphoribosyltransferase
NF-κB	Nuclear Factor Kappa-Light-Chain-Enhancer of Activated B cells
NK	Natural Killer (cell)
NMN	Nicotinamide Mononucleotide
NMNH	Reduced Nicotinamide Mononucleotide
NMNAT	Nicotinamide Mononucleotide Adenylyltransferase
NR	Nicotinamide Riboside
NRH	Dihydronicotinamide Riboside
NRK	Nicotinamide Riboside Kinase
NSAID	Non-Steroidal Anti-Inflammatory Drug
OSKM	Oct4, Sox2, Klf4, c-Myc
PARP	Poly(ADP-Ribose) Polymerase
PGC-1α	Peroxisome Proliferator-Activated Receptor Gamma Coactivator 1-alpha
PI3K	Phosphoinositide 3-Kinase
PKB/AKT	Protein Kinase B
RCT	Randomized Controlled Trial
ROS	Reactive Oxygen Species
SASP	Senescence-Associated Secretory Phenotype
SIRT	Sirtuin
SIRT1	Sirtuin 1
SkQ1	10-(6′-Plastoquinonyl)decyltriphenylphosphonium
STAC	Sirtuin-Activating Compound
TAME	Targeting Aging with Metformin (trial)
TNF-α	Tumor Necrosis Factor-alpha
TSC2	Tuberous Sclerosis Complex 2
ULK1	Unc-51-like Kinase 1
WHO	World Health Organization
